# Phosphorylated Heat Shock Protein 20 (HSPB6) Regulates Transforming Growth Factor-α-Induced Migration and Invasion of Hepatocellular Carcinoma Cells

**DOI:** 10.1371/journal.pone.0151907

**Published:** 2016-04-05

**Authors:** Rie Matsushima-Nishiwaki, Hidenori Toyoda, Tomoaki Nagasawa, Eisuke Yasuda, Naokazu Chiba, Seiji Okuda, Atsuyuki Maeda, Yuji Kaneoka, Takashi Kumada, Osamu Kozawa

**Affiliations:** 1 Department of Pharmacology, Gifu University Graduate School of Medicine, Gifu, Japan; 2 Department of Gastroenterology, Ogaki Municipal Hospital, Ogaki, Gifu, Japan; 3 Department of Radiological Technology, Suzuka University of Medical Science, Suzuka, Mie, Japan; 4 Department of Medical Technology, Ogaki Municipal Hospital, Ogaki, Gifu, Japan; 5 Department of Surgery, Ogaki Municipal Hospital, Ogaki, Gifu, Japan; The University of Hong Kong, CHINA

## Abstract

Human hepatocellular carcinoma (HCC) is one of the major malignancies in the world. Small heat shock proteins (HSPs) are reported to play an important role in the regulation of a variety of cancer cell functions, and the functions of small HSPs are regulated by post-translational modifications such as phosphorylation. We previously reported that protein levels of a small HSP, HSP20 (HSPB6), decrease in vascular invasion positive HCC compared with those in the negative vascular invasion. Therefore, in the present study, we investigated whether HSP20 is implicated in HCC cell migration and the invasion using human HCC-derived HuH7 cells. The transforming growth factor (TGF)-α-induced migration and invasion were suppressed in the wild-type-HSP20 overexpressed cells in which phosphorylated HSP20 was detected. Phospho-mimic-HSP20 overexpression reduced the migration and invasion compared with unphosphorylated HSP20 overexpression. Dibutyryl cAMP, which enhanced the phosphorylation of wild-type-HSP20, significantly reduced the TGF-α-induced cell migration of wild-type HSP20 overexpressed cells. The TGF-α-induced cell migration was inhibited by SP600125, a c-Jun N-terminal kinases (JNK) inhibitor. In phospho-mimic-HSP20 overexpressed HuH7 cells, TGF-α-stimulated JNK phosphorylation was suppressed compared with the unphosphorylated HSP20 overexpressed cells. Moreover, the level of phospho-HSP20 protein in human HCC tissues was significantly correlated with tumor invasion. Taken together, our findings strongly suggest that phosphorylated HSP20 inhibits TGF-α-induced HCC cell migration and invasion via suppression of the JNK signaling pathway.

## Introduction

The hepatocellular carcinoma (HCC) is one of the major malignancies in the world, and the third cause of cancer-related death [1,2]. Liver carcinogenesis is generally associated with chronic hepatitis B, chronic hepatitis C, alcohol abuse, aflatoxin exposure or nonalcoholic steatohepatitis (NASH) [2]. Chronic hepatitis C in combination with alcohol abuse is the primary risk factor of HCC, and NASH is also recently emerged as a relevant risk factor in developed countries [1,2]. The five-year survival rate of HCC is 30–40%, and recurrence and metastasis frequently occurs even after surgical resection [3]. HCC is known as an aggressive tumor for its propensity to directly invade the portal and hepatic veins [4], and extrahepatic tumor spread of HCC is frequently observed [5,6]. Circulating HCC cells are recognized as the cause for recurrence, invasiveness, and metastasis [7–9]. Accumulating evidence indicates that transforming growth factor (TGF)-α is implicated in migration and invasion of cancer cells including HCC [10–12]. It has been shown that the intracellular signaling of TGF-α through activation of epidermal growth factor (EGFR), a receptor for TGF-α, enhances the movement of HCC cells [12]. The levels of TGF-α are reportedly increased in metastatic liver tumor [10]. However, the exact mechanism behind TGF-α-effects on HCC metastasis remains to be clarified.

Heat shock protein 20 (HSP20/ HSPB6) is one of the small HSP family (HSPB) with monomer molecular mass in the range from 15 to 30 kDa [13,14]. Ten HSPBs (HSPB1 to HSPB10) have been identified in the human genome based on sequence homology, and are characterized by the presence of a highly conserved domain “α-crystallin domain” [13,14]. Among HSPBs, HSP27 (HSPB1), αB-crystallin (HSPB5), HSP22 (HSPB8) and HSP20 (HSPB6) are ubiquitously expressed in a variety type of organs including liver [13,14]. HSPs are well recognized to act as molecular chaperones and HSP20 also has a molecular chaperone activity. It is generally known that HSP expression is mediated by heat shock factor (HSF)-1 [15]. Although some factors might physiologically affect HSP20 expression, the expression of HSP20 is not induced by heat or chemical stress, and does not seem to depend on the action of HSF-1 [13]. Accumulating evidence suggests that HSP20 is implicated in multiple physiological and pathological processes, such as regulation of smooth muscle relaxation, myocardial infarction, and Alzheimer’s disease [13,14,16,17]. We have previously reported that HSP20 suppresses the aggregation and activation of human platelets extracellularly [18,19]. In HCC, we have demonstrated that the HSP20 expression levels are inversely correlated with the TNM stage [20]. The TNM classification records the primary and regional nodal extent of the tumor, and the metastasis. In addition, we showed that HSP20 stimulates apoptosis of HCC cells by direct interaction with Bax, and that HSP20 down-regulates tumor necrosis factor (TNF)-α-stimulated nuclear factor-κB (NF-κB) signaling pathway by suppression of inhibitor κB kinase (IKK)-α expression in human HCC [21,22]. Furthermore, HSP20 reduces TGF-α-stimulated HCC cell growth by suppressing the mitogen-activated protein kinase (MAPK) family, including c-Jun N-terminal kinase (JNK) and the phosphoinositide 3-kinase (PI3K)/AKT pathway [23,24].

The functions of HSPBs are regulated by post-translational modification, such as phosphorylation [13]. As for HSP20 (HSPB6), it has been shown that phosphorylated HSP20 (serine 16) is catalyzed by cyclic nucleotide-dependent protein kinases, such as protein kinase A and protein kinase G, and regulates the interaction of myosin with actin, which correlates with the relaxation of trachealis and carotid artery smooth muscle [13]. Tight interaction of phosphorylated HSP20 with universal adapter protein 14-3-3 reportedly inhibits the interaction of phosphorylated cofilin with 14-3-3, and induces depolymerization of actin filaments [13]. Moreover, it has been reported that phospho-mimicking mutant of HSP20 decreases apoptosis of cardiomyocytes, resulting in the protection against ischemia/reperfusion injury [13]. However, the exact roles of phosphorylated HSP20 have not yet been fully understood.

Regarding HSPB-effects on cell migration and invasion, HSP27 reportedly increases the cell migration of cancers, such as prostate cancer and HCC [25,26]. We have reported that HSP22 acts as a positive regulator in the TGF-α-stimulated migration of ovary cancer cells [27]. Moreover, HSP20 reportedly promotes human umbilical vein endothelial cells (HUVECs) migration in cardiac angiogenesis [28]. We previously showed that the expression levels of HSP20 inversely correlated with the presence of the microvascular invasion of human HCC [20]. However, the involvement of HSP20 in the migration and invasion of HCC cells has not yet been clearly elucidated. The aim of this study was to investigate the roles of HSP20 and its phosphorylation status in TGF-α-stimulated migration of HCC cells and the invasion. We herein demonstrate that phosphorylated HSP20 functions as a suppressive regulator in HCC cell migration and the invasion.

## Materials and Methods

### Antibodies and Chemicals

HSP20 antibodies were purchased from Enzo Life Sciences Inc. (Farmingdale, NY). Antibodies recognizing phosphorylated serine 16 in HSP20 were constructed as described previously [29], or obtained from Abcam plc (Cambridge, UK). Antibodies against phospho-JNK and phospho-EGFR (Y1068) were purchased from Cell Signaling Technology, Inc. (Danvers, MA). Glyceraldehyde-3-phosphate dehydrogenase (GAPDH) antibodies were purchased from Santa Cruz Biotechnology Inc. (Santa Cruz, CA). Recombinant human TGF-α was obtained from R&D systems Inc. (Minneapolis, MN). SP600125 was purchased from Calbiochem-Novabiochem Co. (La Jolla, CA). SP600125 was dissolved in dimethyl sulfoxide. The maximum concentration of dimethyl sulfoxide was 0.1%, which did not affect the cell migration assay or Western blot analysis. N^6^,2’-O-dibutyryladenosine 3,5’-cyclic monophosphate (dibutyryl cAMP) was purchased from Sigma-Aldrich Co. (St Louis, MO). The BCA protein assay kit was obtained from Thermo Fisher Scientific Inc. (Waltham, MA). All other materials and chemicals were obtained from commercial sources.

### Plasmids

Wild-type (WT) human HSP20 cDNA (clone ID 6074542), was obtained as previously described [23]. Human HSP20 mutants, unphosphorylated type (SA) or phospho-mimic type (SD), were constructed using the following primers: 5’-TGG CTG CGC CGC GCC GCG GCC CCG TTG CTC GGA-3 (forward), 5’-TCC GAG CAA CGG GGC CGC GGC GCG GCG CAG CCA-3’ ‘ (reverse) for SA; and 5’-TGG CTG CGC CGC GCC GAC GCC CCG TTG CTC GGA-3 (forward), 5’-TCC GAG CAA CGG GGC GTC GGC GCG GCG CAG CCA-3’ ‘ (reverse) for SD. The primers for SA and SD, designed manually by replacing the nucleotides in the middle of the primer sequence, have alanine and aspartate in the place of the serine codon, respectively. The cDNA construct of WT human HSP20 in pcDNA3.1(+) was used as the template for generating the mutants using the Quick Change II XL site-directed mutagenesis kit (Stratagene, La Jolla, CA) following the manufacturer’s protocol. PCR amplification was performed with an initial denaturation of 95°C for 1 min, followed by 21 cycles of 95°C for 50 s, 50°C for 50 s, and 68°C for 9 min followed by an overall extension at 68°C for 7 min. The amplicons were digested with *DpnI* for 1 h at 37°C, and the products were transformed into XL-10 Gold competent *Escherichia coli* cells. The transformed cells were selected on Luria vertani broth (LB) agar plates containing 100 μg/ml ampicillin. The mutant constructs were validated with restriction digestion and DNA sequence analysis.

### Cell Culture and Establishment of Stably Transfected Cell Line

Human HCC-derived HuH7 cells were obtained from the Health Science Reseach Resources Bank (Tokyo, Japan). The HuH7 cells were maintained in Roswell Park Memorial Institute (RPMI) 1640 (Sigma-Aldrich Co.) medium supplemented with 10% fetal calf serum (FCS) (Hyclone Corp., Logan, UT).

The stably WT-HSP20 overexpressed and control empty vector transfected HuH7 cells have been established as previously described in [23]. The stably mutants (SA or SD)-HSP20 overexpressed HuH7 cells were established by means of Tet-Off^™^ gene expression systems (Clontech Laboratories Inc., Palo Alto, CA) according to the manufacturer’s instructions. Induction of the mutants-HSP20 protein expression in the HSP20 overexpressed HuH7 cells can be controlled by the presence of doxycycline (Sigma-Aldrich, Co.). The WT- and the mutants-HSP20 overexpressed HuH7 cells, and the control empty vector-transfected HuH7 cells were maintained in RPMI1640 supplemented with 10% FCS, 200 μg/ml G418 (Life Technologies Co.), 100 μg/ml hygromycin B (Merck KGaA Co. Darmstadt, Germany) and 1 μg/ml doxycycline.

### Assay for Cell Migration and Cell Invasion

Cell migration and cell invasion were assessed by using Boyden chamber (polycarbonate membrane with 8-μm pores, Transwell^®^, Corning Costar Co., Cambridge, MA) and Corning^®^ BioCoat^™^ matrigel invasion chamber (8-μm pores, Corning Inc., Tewksbury, MA), respectively. The cells were trypsinized and seeded (1×10^5^ cells/well) onto the upper chamber in serum-free RPMI1640. When indicated, the cells were pretreated with dibutyryl cAMP or SP600125 in the lower chamber for 1 h at 37°C. Three ng/ml TGF-α was then added to the lower chamber and incubated for 24 h at 37°C for migration assay. For invasion assay, 10 ng/ml TGF-α was added to the lower chamber and incubated for 48 h at 37°C. After incubation, the cells on the upper surface of the membrane were mechanically removed. The migrated or invaded cells adherent to the underside of the membrane were fixed with 4% paraformaldehyde and stained with 4’,6-diamidino-2-phenylindole (DAPI) solution. The cells were then photographed and counted using fluorescent microscopy at a magnification of 20× by counting the stained cells from three randomly chosen high-power fields.

### Western Blot Analysis

For Western blot analysis, the cells were seeded into 90-mm (7×10^5^ cells/dish) diameter dishes. After 3 days, the medium was exchanged for serum-free RPMI1640, and the cells were then stimulated by 30 ng/ml TGF-α or vehicle after 24 h. When indicated, the cells were treated with the indicated concentrations of dibutyryl cAMP for 1 h. After stimulation, The cells were washed twice with phosphate-buffered saline (PBS) and then lysed and sonicated in lysis buffer containing 62.5 mM Tris-HCl (pH 6.8), 2% sodium dodecyl sulfate (SDS), 50 mM dithiothreitol, and 10% glycerol. SDS-polyacrylamide gel electrophoresis (PAGE) was performed by the method of Laemmli [30]. Snap-frozen human HCC tissues were also lysed in the lysis buffer for Western blot analysis as previously [23]. A Western blot analysis was performed as described previously [23,24] using phospho-HSP20 antibodies, HSP20 antibodies, phospho-JNK antibodies, phospho-EGFR antibodies and GAPDH antibodies, with peroxidase-labeled anti-rabbit IgG antibodies (Cell Signaling Technology, Inc.) as secondary antibodies. The peroxidase activity on polyvinylidene difluoride membrane was visualized on X-ray film using the ECL Western blotting detection system (GE Healthcare, Waukesha, WI). Densitometric analysis was performed using scanner and image analysis software (ImageJ, version 1.48). The background-subtracted signal intensity of each phosphorylation signal was normalized to the respective total protein or GAPDH signals, and plotted.

### Tissue Specimens

HCC tissues were obtained by surgical resection from patients in the Department of Surgery, Ogaki Municipal Hospital (Gifu, Japan) according to a protocol approved by the committee for the conduct of human research at Ogaki Municipal Hospital and at Gifu University Graduate School of Medicine. Written informed consent was obtained from all of the patients. Exclusion criteria were death by the disease besides HCC and over the average ± 2 SD of phospho-HSP20, total-HSP20, and GAPDH protein levels.

### Statistical Analysis

The data are expressed as the means ± SD. The statistical significance of the data from the cell culture experiments and the HCC tissues experiments were analyzed using the student’s t-test and Fisher’s exact test, respectively. The values of *P* < 0.05 were considered to be statistically significant. Each cell culture experiment was repeated three times with similar results.

## Results

### Expression of HSP20 in the WT-, SA- and SD-HSP20 Stably Transfected HuH7 Cells

**Fig 1:**
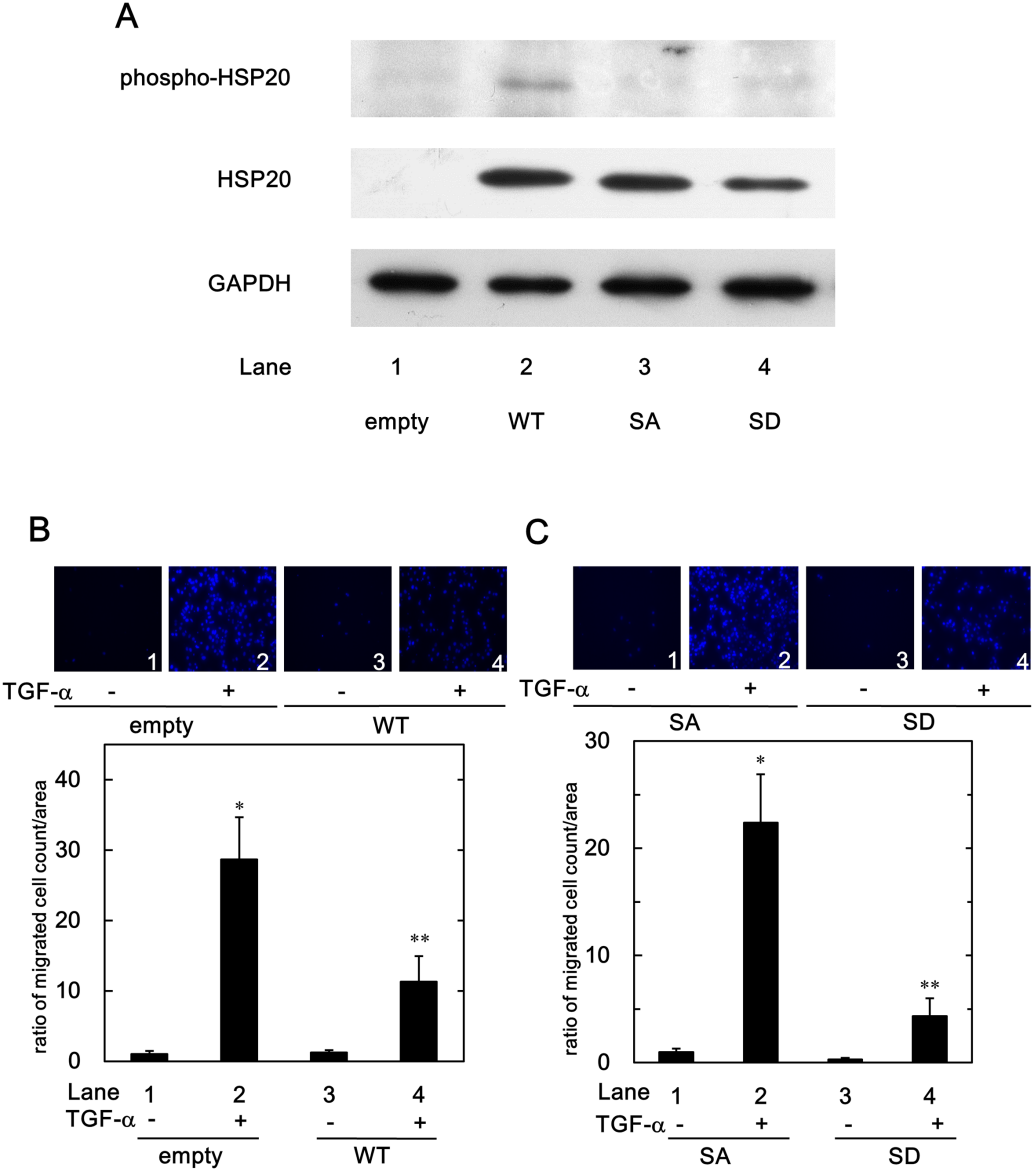
Phosphorylated HSP20 levels in the wild-type- and the mutants-HSP20 transfected HuH7 cells, and suppression of the TGF-α-induced HuH7 cell migration by HSP20. (A), The protein expression of the wild-type- and the mutants-HSP20 in the HuH7 cells were determined by Western blotting using HSP20 antibodies and phospho-HSP20 (serine 16) antibodies. HuH7 cells were stably transfected either with control empty-, wild-type-HSP20-expressing (WT), its alanine mutant-expressing (SA) or its aspartate mutant-expressing (SD) vectors. The empty vector- or the WT-HSP20 transfected HuH7 cells (B), or the SA- or the SD-HSP20 overexpressed HuH7 cells (C) were stimulated by 3 ng/ml of TGF-α or vehicle for 24 h. The migrated cells were fixed with paraformaldehyde and stained with DAPI for the nucleus (blue signal). The cells were photographed by fluorescent microscopy at a magnification of 20× (upper panel). The migrated cell numbers were counted and plotted as the ratio to the mean of migrated numbers of the control empty vector-transfected cells (B), or the SA-HSP20 overexpressed cells (C) without TGF-α stimulation (lower panel). Each value represents the means ± SD (n = 3). *P<0.05, compared to the value of lane 1. **P<0.05, compared to the value of lane 2.

Although the HSP20 protein expressed in the tumor tissues of human HCC, we previously showed that the HSP20 protein does not express in the HCC cell lines [20,23,24]. To investigate the roles of HSP20 in HCC cells, we already have established WT-HSP20 and empty vector stably transfected human HCC-derived HuH7 cells as previously described in [23,24]. HSP20 has been reported to be phosphorylated at serine 16 residue by cyclic nucleotide-dependent protein kinases [13]. Therefore, in order to clarify the effect of phosphorylated HSP20 on HCC cells, we established the stably unphosphorylated type mutant (SA)- or the stably phospho-mimic type mutant (SD)-HSP20 cDNAs transfected HuH7 cells in this study. A Western blot analysis (Fig 1A) demonstrated that HSP20 was overexpressing in the SA- and SD-HSP20 transfected HuH7 cells as well as in the WT-HSP20 transfected cells as previously described [23]. Although weak reaction, anti-phospho-HSP20 antibodies detected the phospho-HSP20 protein in WT-HSP20 transfected HuH7 cells (Fig 1A, lane 2). On the other hand, the overexpressed HSP20 protein in SA- or SD-HSP20 transfected cells did not react with the phospho-HSP20 antibodies (Fig 1A, lanes 3 and 4, respectively), although it is generally recognized that the mutation of serine to aspartate mimics the phosphorylated type of protein [31].

### Effect of HSP20 on TGF-α-induced HuH7 Cell Migration

It has been shown that the TGF-α is frequently oversynthesized in human HCC, especially in metastatic tumors [10,32,33]. TGF-α has been reported to promote the migration of human HCC. In addition, the activation of EGFR, the receptor of TGF-α, plays an important role in HCC cell movement [12]. We found that 3 ng/ml TGF-α significantly stimulated the HuH7 cell migration (data not shown). Therefore, we first investigated the involvement of HSP20 in TGF-α-induced migration of human HCC cells by using the WT-HSP20 overexpressed HuH7 cells.

As shown in Fig 1B, the TGF-α-induced migration of WT-HSP20 overexpressed HuH7 cells was significantly reduced in comparison with that of the empty vector-transfected cells.

### Effect of Phosphorylated HSP20 on TGF-α-induced HuH7 Cell Migration

It is well known that phosphorylation is the most versatile post-translational modification, and it modifies the protein functions [34,35]. We found the phosphorylated HSP20 in WT-HSP20 overexpressed HuH7 cells was detected as shown above (Fig 1A, lane 2). We next investigated the effect of HSP20 phosphorylation on TGF-α-induced migration of HuH7 cells. The TGF-α-stimulated migrated numbers of phospho-mimic SD-HSP20 overexpressed HuH7 cells were significantly suppressed compared with the unphosphorylated type SA-HSP20 overexpressed HuH7 cells (Fig 1C, panel and lane 4 compared to panel and lane 2). Phosphorylated HSP20 might play a suppressive role in the TGF-α-induced migration of HCC cells.

### HSP20 Phosphorylation in WT-HSP20 Overexpressed HuH7 Cells by dibutyryl cAMP

**Fig 2:**
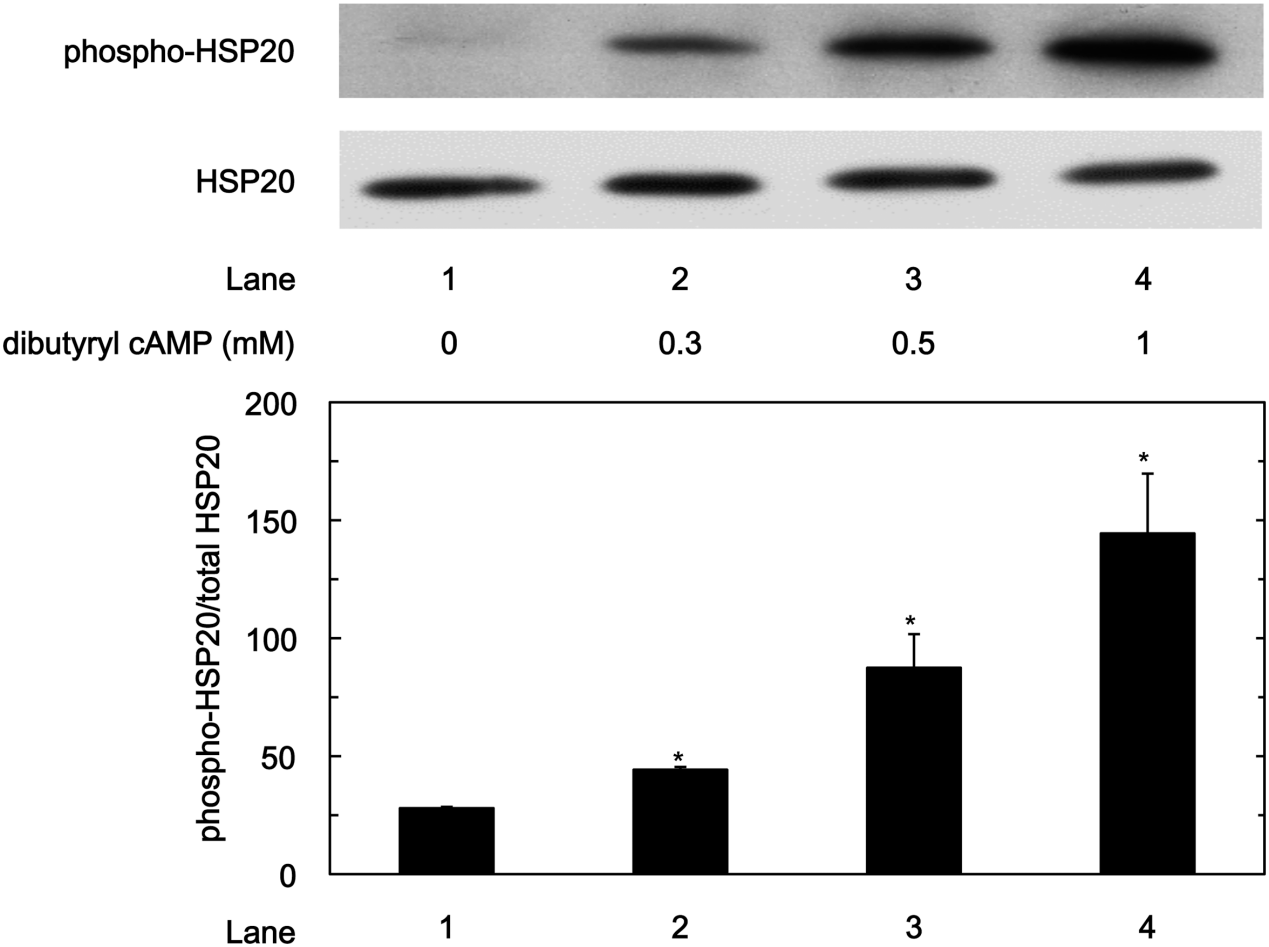
Phosphorylation of HSP20 by dibutyryl cAMP in HuH7 cells. The WT-HSP20 overexpressed HuH7 cells were treated with the indicated concentrations of dibutyryl cAMP for 1 h. After treatment, the cells were harvested, and Western blot analysis was performed to determine the levels of phosphorylated HSP20. The lower bar graph shows the quantification data for the relative levels of phospho-HSP20 after normalization with the total HSP20 levels as determined by densitometry analysis. Each value represents the means ± SD (n = 3). *P<0.05, compared to the value of lane 1.

We examined whether the HSP20 protein in the WT-HSP20 overexpressed HuH7 cells could be furthermore phosphorylated by dibutyryl cAMP or 8-bromo guanosine 3’,5’-cyclic monophosphate (8-bromo cGMP). The levels of phosphorylated HSP20 at serine 16 in the WT-HSP20 overexpressed HuH7 cells was significantly upregulated by dibutyryl cAMP treatment in a dose-dependent manner between 0.3 mM and1 mM (Fig 2). On the contrary, 8-bromo cGMP did not increase the phosphorylation levels of HSP20 even at 1 mM (data not shown).

### Effect of Dibutyryl cAMP Treatment to WT-HSP20 Overexpressed HuH7 Cells on TGF-α-induced HuH7 Cell Migration

**Fig 3:**
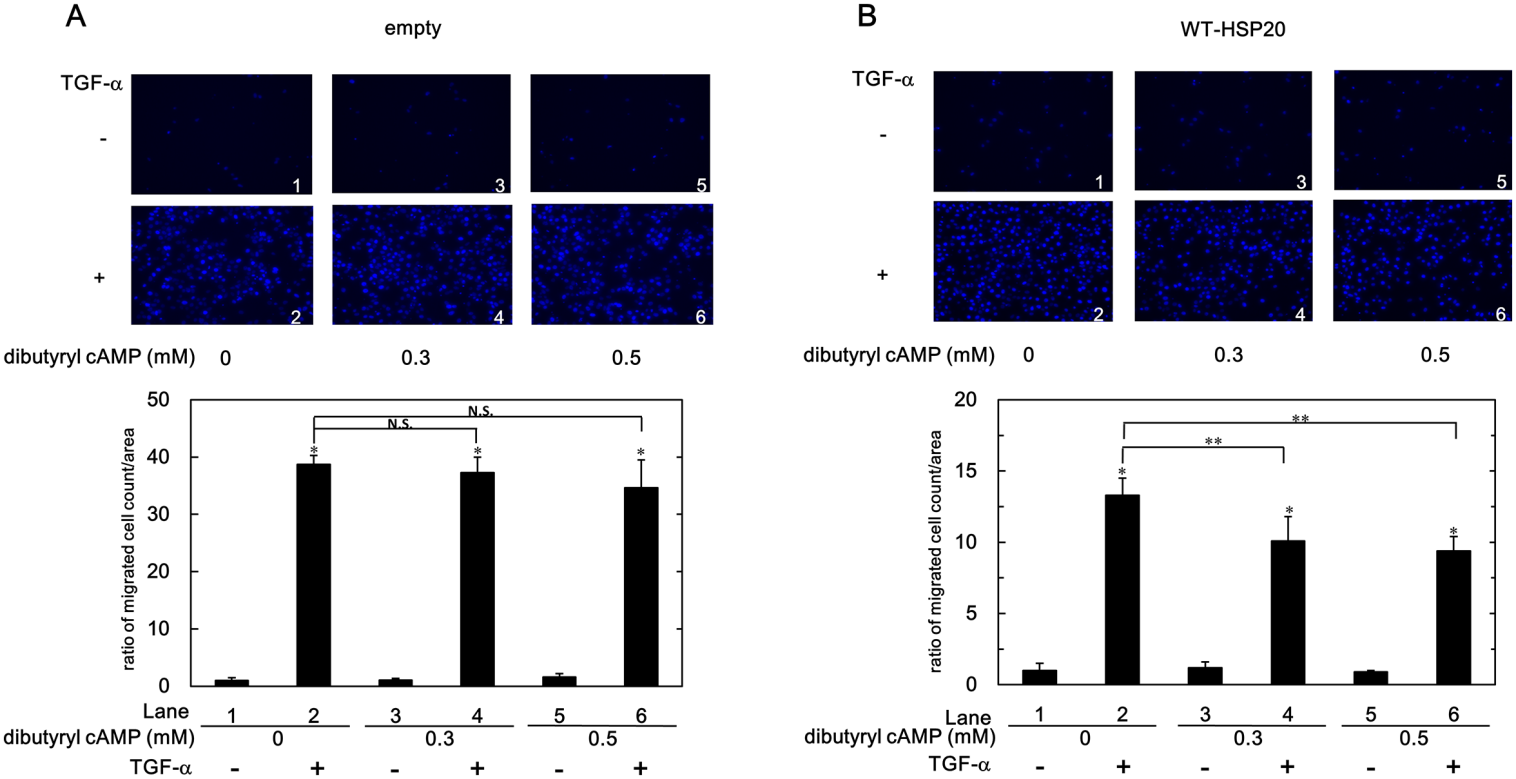
Dibutyryl cAMP suppressed the TGF-α-induced cell migration of WT-HSP20 overexpressed HuH7 cells. The empty vector- (A) or the WT-HSP20- (B) transfected HuH7 cells were pretreated with the indicated concentrations of dibutyryl cAMP for 1 h, and then stimulated by 3 ng/ml of TGF-α or vehicle for 24 h. The migrated cells were fixed with paraformaldehyde and stained with DAPI for the nucleus (blue signal). The cells were photographed by fluorescent microscopy at a magnification of 20× (upper panel). The migrated cell numbers were counted and plotted as the ratio to the mean of migrated numbers of the cells stimulated by vehicle without dibutyryl cAMP treatment (lower panel). Each value represents the means ± SD (n = 3). *P<0.05, lanes 2, 4 and 6 compared to the values of lanes 1, 3, and 5, respectively. **P<0.05. lanes 4 and 6 compared to the value of lane 2.

We next investigated the effect of dibutyryl cAMP treatment on TGF-α-induced HuH7 cell migration. Pretreatment of dibutyryl cAMP significantly suppressed the migrated numbers of the TGF-α-stimulated WT-HSP20 overexpressed HuH7 cells, even at 0.3 mM (Fig 3B). On the other hand, dibutyryl cAMP pretreatment did not show any effect on the migrated numbers of the TGF-α-stimulated empty vector-transfected HuH7 cells (Fig 3A). Phosphorylated HSP20 in WT-HSP20 overexpressed HuH7 cells might have a suppressive role in the TGF-α-induced cell migration.

### Effect of HSP20 on TGF-α-induced HuH7 Cell Invasion

**Fig 4:**
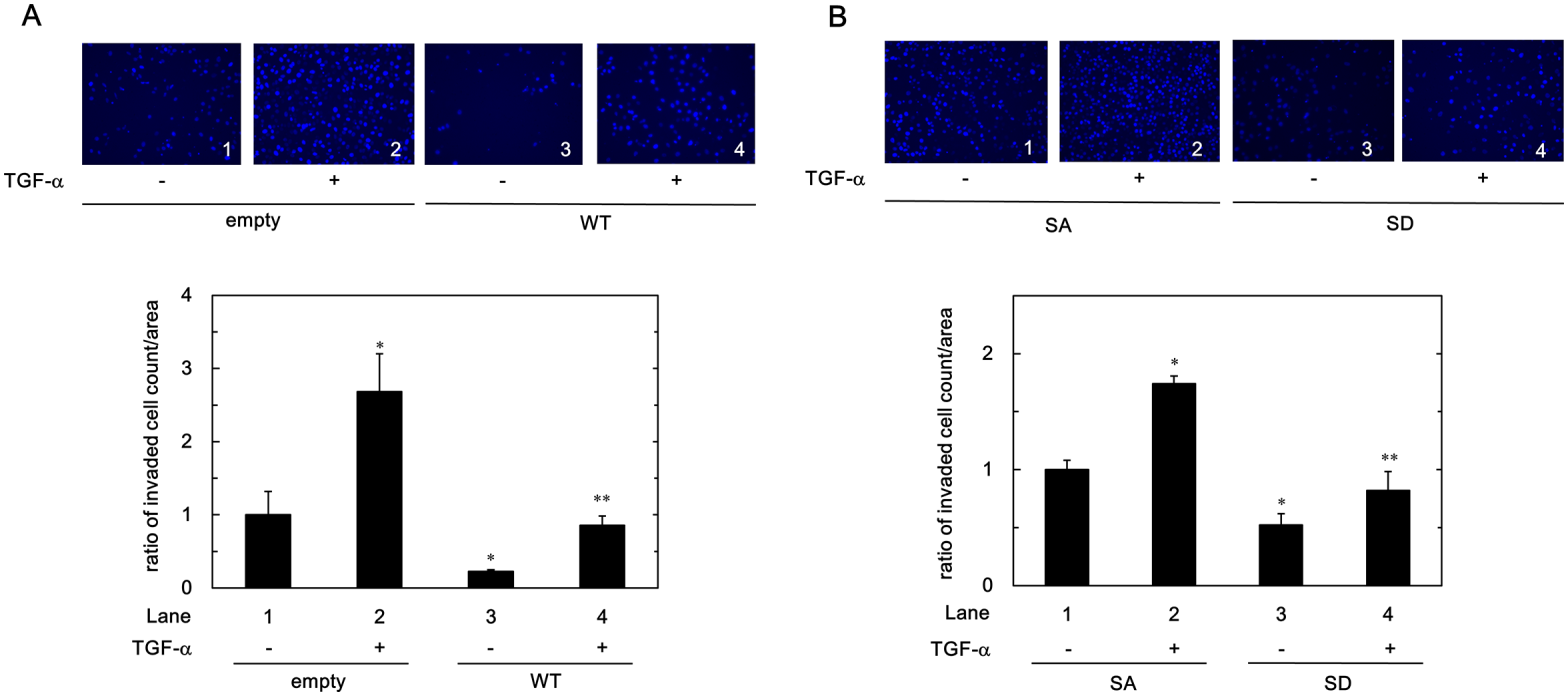
Wild type and phospho-mimic HSP20 suppressed the TGF-α-induced HuH7 cell invasion. The empty vector- or the WT-HSP20- transfected (A), or the SA- or the SD-HSP20 overexpressed (B) HuH7 cells were stimulated by 10 ng/ml of TGF-α or vehicle for 48 h. The invaded cells were fixed with paraformaldehyde and stained with DAPI for the nucleus (blue signal). The cells were photographed by fluorescent microscopy at a magnification of 20× (upper panel). The invaded cell numbers were counted and plotted as the ratio to the mean of invaded numbers of the empty vector-transfected (A) or the SA-HSP20 overexpressed (B) HuH7 cells without TGF-α stimulation (lower panel). Each value represents the means ± SD (n = 3). *P<0.05, compared to the value of lane 1. **P<0.05, compared to the value of lane 2.

In our previous study [20], we showed that the presence of the microvascular invasion of human HCC is correlated with the levels of HSP20 protein expression. Therefore, we next investigated the role of HSP20 on TGF-α induced invasion of HuH7 cells. After 48 h incubation, the invaded numbers of the empty vector-transfected HuH7 cells were significantly induced by TGF-α stimulation (Fig 4). However, the invaded numbers of the WT-HSP20 transfected HuH7 cells were significantly suppressed compared with empty vector-transfected HuH7 cells both in the presence or the absence of TGF-α stimulation (Fig 4A, panels and lanes 3 and 4 compared to panels and lanes 1 and 2, respectively).

### Effect of Phosphorylated HSP20 on TGF-α-induced HuH7 Cell Invasion

Because of phosphorylated HSP20 showed the suppressive effect to the TGF-α-induced migration of HCC cells (Figs 1C and 3), we examined whether phosphorylated HSP20 affects HCC cell invasion using mutant HSP20 overexpressed HuH7 cells. The invaded numbers of the SD-HSP20 overexpressed HuH7 cells were significantly suppressed compared with SA-HSP20 overexpressed HuH7 cells both in the presence or the absence of TGF-α stimulation (Fig 4B, panels and lanes 3 and 4 compared to panels and lanes 1 and 2, respectively). Phosphorylated of HSP20 might suppress not only the migration of the HCC cells but also the invasion.

### Effect of JNK Inhibitor on TGF-α-induced HuH7 Cell Migration

**Fig 5:**
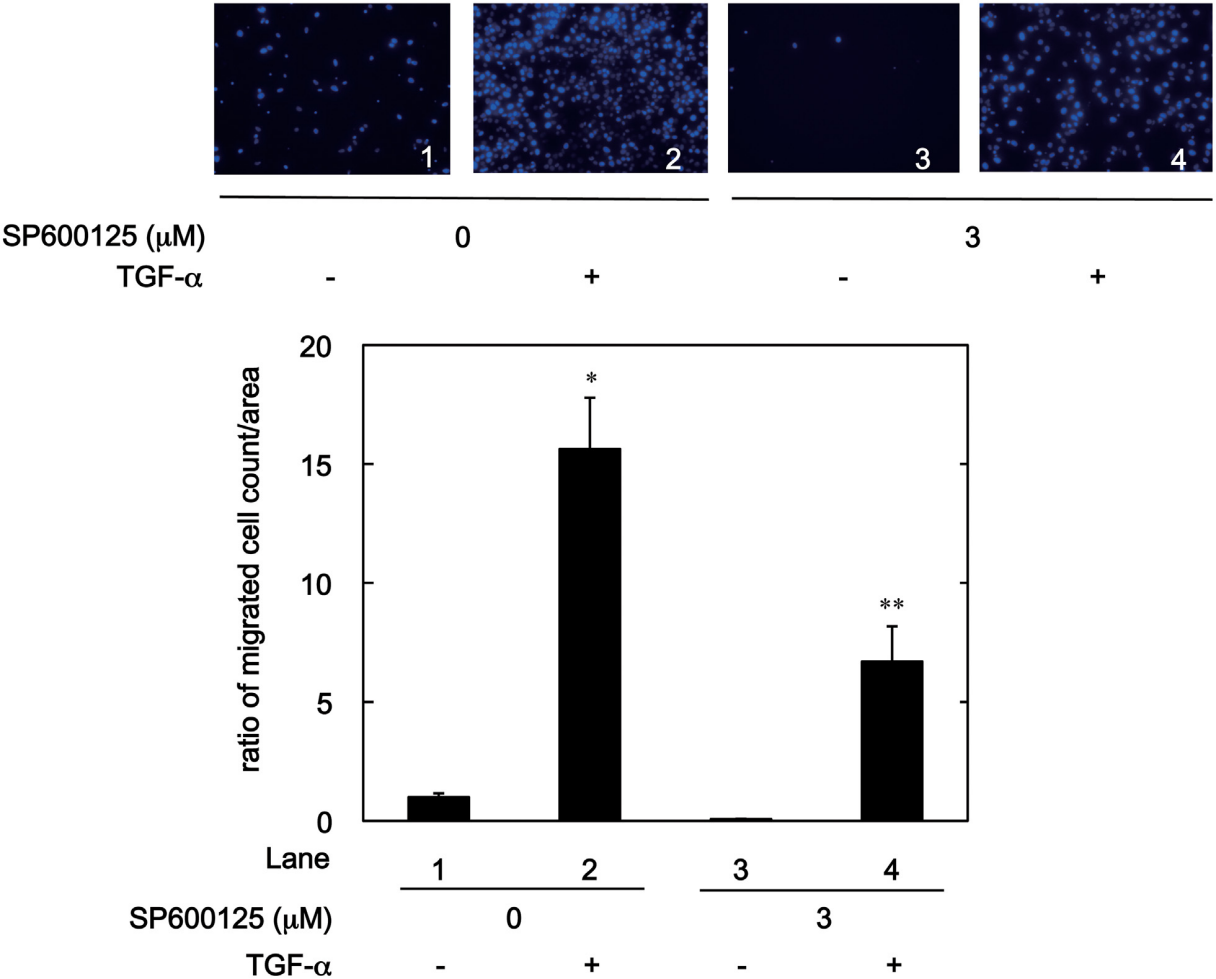
SP600125 decreased the TGF-α-induced HuH7 cell migration. The HuH7 cells were pretreated with 3 μM of SP600125 or vehicle for 1 h, and then stimulated by 3 ng/ml of TGF-α or vehicle for 24 h. The migrated cells were fixed with paraformaldehyde and stained with DAPI for the nucleus (blue signal). The cells were photographed by fluorescent microscopy at a magnification of 20× (upper panel). The migrated cell numbers were counted and plotted as the ratio to the mean of migrated numbers of the cells stimulated by vehicle without SP600125 treatment (lower panel). Each value represents the means ± SD (n = 3). *P<0.05, compared to the value of lane 1. **P<0.05, compared to the value of lane 2.

MAPKs have a central role in HCC development [33]. Especially, accumulating evidence suggests that the JNK pathway among the MAPK family is required for the HCC cell migration [33]. We have previously shown that HSP20 has an inhibitory role in the TGF-α-induced JNK activation in the WT-HSP20 overexpressed HuH7 cells [23] As shown in Fig 5, we found that SP600125, a JNK inhibitor [36], significantly reduced the TGF-α-induced migration of naive HuH7 cells (panel and lane 4 compared to panel and lane 2).

### Effect of Phosphorylated HSP20 on the TGF-α-induced JNK Activation

**Fig 6:**
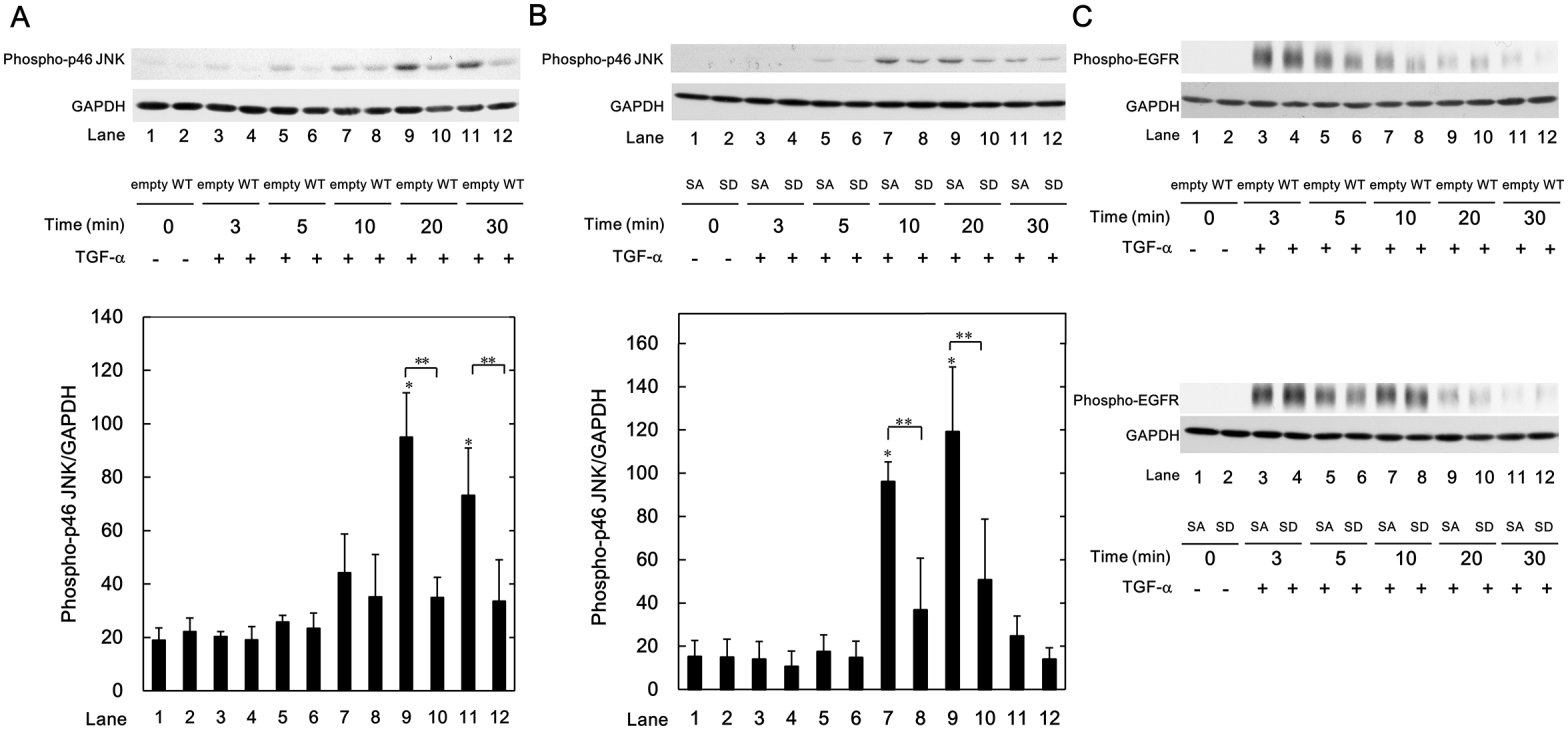
Phospho-mimic HSP20 attenuated the TGF-α-induced JNK signaling in HuH7 cells. The empty vector-, WT-, SA- or SD-HSP20 transfected HuH7 cells were stimulated by 30 ng/ml of TGF-α for the indicated times. The cells were then harvested, and Western blot analysis was performed to determine the levels of phospho-p46 JNK (A and B) and phospho-EGFR (C). The lower bar graph (A and B) shows the quantification data for the relative levels of phospho-p46 JNK after normalization with GAPDH levels as determined by densitometry analysis. Each value represents the means ± SD (n = 3). *P<0.05, compared to the value of lane 1. **P<0.05, lanes 8 and 10, compared to the values of lanes 7 and 9, respectively.

Previously, we reported that HSP20 suppresses the TGF-α-induced HuH7 cell proliferation via the JNK signaling [23]. In addition, TGF-α-induced levels of phospho-JNK in the WT-HSP20 overexpressed HuH7 cells are weaker than those in the empty vector-transfected cells [23]. We confirmed the phenomenon also in the present study (Fig 6A). Therefore, to investigate the effect of phosphorylated HSP20 on the TGF-α-induced JNK activation, we compared the TGF-α-induced JNK activation in the SD-HSP20 overexpressed HuH7 cells to that in the SA-HSP20 overexpressed HuH7 cells. As shown in Fig 6B, TGF-α-induced levels of phospho-JNK in the SD-HSP20 overexpressed HuH7 cells were significantly suppressed compared with those in the SA-HSP20 overexpressed HuH7 cells at 10 and 20 min after TGF-α stimulation (lanes 8 and 10 compared to lanes 7 and 9, respectively). Phosphorylated HSP20 might suppress TGF-α-induced JNK activation in HCC cells.

It is generally known that TGF-α is a ligand for EGFR, and the JNK signaling is one of the main pathways in the EGFR activation [32]. However, the TGF-α-induced levels of phosphorylated EGFR did not show any change either by the WT-HSP20 expression compared with the empty vector transfection, or by the SD-HSP20 overexpression compared with the SA-HSP20 overexpression in HuH7 cells (Fig 6C).

### Relationship Between the Levels of Phospho-HSP20 Protein and Tumor Invasion in Human HCC Tissues

Because of the HSP20 phosphorylation suppressed HuH7 cell migration and invasion (Figs 1C, 3 and 4B), and HSP20 expression levels reportedly correlate with TGF-α-induced JNK activation in human HCC tissues [23], we next observed the relationship between phospho-HSP20 protein level in human HCC tissues and tumor invasion. Phospho-HSP20 protein levels of HCC tissues were determined by Western blot analysis. The clinical and pathological characteristics of the patients with HCC are shown in Table 1. In order to be compared with the invasion, the 36 patients were divided by the median of phospho-HSP20/GAPDH (0.279 A.U.) into either phospho-HSP20/GAPDH low quantity level group (< 0.279 A.U.) or phospho-HSP20/GAPDH high quantity level group (≥ 0.279 A.U.). As shown in Table 2, phospho-HSP20 levels were significantly correlated with HCC tumor invasion.

**Table 1:** The clinical and pathological characteristics of patients with HCC.

**Gender**	Male; n = 29	Female; n = 7
**Underlying disease**	Liver cirrhosis; n = 13	Chronic hepatitis; n = 21
	Fatty liver; n = 1	Normal liver; n = 1
**Etiology of liver disease**	HBV; n = 8	HCV; n = 18
	Other; n = 10	
**Number of tumors**	Solitary; n = 28	Multiple; n = 8
**Tumor size (mm)**	<20; n = 4	20–50; n = 26
	>50; n = 6	
**Vascular invasion**	Negative; n = 29	Positive; n = 7
**Tumor stage**	I; n = 7	II; n = 15
	III; n = 12	IV; n = 2
**Histological classification**	Well; n = 7	Moderately; n = 29
**(differentiation)**	Poorly; n = 0	

**Table 2:** Relationship between the protein level of phospho-HSP20 in HCC and tumor invasion.

	phospho-HSP20/GAPDH (A.U.)		
	<0.279	≥0.279	P*
**Invasion**			
Negative	12	17	0.0438
Positive	6	1	

*Fisher’s exact test.

## Discussion

The recurrence of HCC is the main cause of death by HCC. It is well recognized that HCC is susceptible to invade portal and hepatic veins, and that development of intrahepatic and extrahepatic tumor spread is frequently observed [4–6]. Therefore, circulating HCC cells are the primary source of the HCC recurrence [9]. TGF-α is secreted by tumor cells, and its amount in HCC patients are significantly higher than healthy human [37]. It has been shown that expression of human TGF-α in fibroblasts obtains an anchorage-independent cell growth activity [38]. We previously demonstrated that the levels of HSP20 in human HCC are inversely correlated with the presence of microvascular invasion [20]. In the present study, therefore, we investigated whether HSP20 regulates the TGF-α-induced migration of HCC cells and invasion. We demonstrated that the TGF-α-induced migration and invasion of WT-HSP20 overexpressed HuH7 cells were markedly suppressed. We found that phosphorylated HSP20 (serine 16) was detected in the WT-HSP20 overexpressed HuH7 cells. Therefore, we investigated the role of phosphorylated HSP20 in the HCC cell migration and the invasion. We showed that TGF-α-induced migration of phospho-mimic SD-HSP20 overexpressed HuH7 cells was remarkably reduced in comparison with the unphosphorylated type of SA-HSP20 overexpressed HuH7 cells. HSP20 at serine 16 is reportedly phosphorylated by the cyclic nucleotide-dependent protein kinase [13], and we found the levels of the phospho-HSP20 in WT-HSP20 transfected HuH7 cells were increased by dibutyryl cAMP. We herein demonstrated that dibutyryl cAMP reduced the TGF-α-induced WT-HSP20 overexpressed HuH7 cells migration, while, it did not show any effect on the TGF-α-induced migration of control empty vector-transfected HuH7 cells. Based on our findings, it is probable that phosphorylated HSP20 has an inhibitory role in the HCC cell migration. Moreover, we showed that TGF-α-induced invasion of phospho-mimic SD-HSP20 overexpressed HuH7 cells was also remarkably suppressed in comparison with the unphosphorylated type of SA-HSP20 overexpressed HuH7 cells. Furthermore, we found that the levels of phospho-HSP20 significantly correlates with tumor invasion in human HCC tissues. Taken our findings into account, it is most likely that phosphorylated HSP20 reduces HCC cell migration and the invasion. Our results may provide the basis for a novel defensive system to HCC metastasis.

It is currently established that the MAPK superfamily is implicated in cell migration, and accumulating evidence suggests that the JNK pathway among the family is essential for cell migration [12,33,39,40]. Inhibition of JNK reportedly suppresses the migration of HCC cells [33,40]. In the present study, we showed that a JNK inhibitor, SP600125 [36], reduced TGF-α-induced migration of HuH7 cells. It has been shown that the activation of the JNK pathway increases the matrix metalloproteases expression, thus enhances human HCC derived HLE cell invasion [33]. We previously reported that WT-HSP20 overexpression suppresses TGF-α-induced JNK activation in HuH7 cells, and there is an inverse correlation between HSP20 protein expression and JNK activity in human HCC tissues [23]. In the present study, we also confirmed that the levels of phospho-JNK were reduced by WT-HSP20 overexpression. Among JNK, JNK1 is especially over-activated in HCC patients [33]. We found that phosphorylated HSP20 was detected in the WT-HSP20 overexpressed HuH7 cells described above in Fig 1A. In the present study, phospho-mimic HSP20 expression markedly suppressed TGF-α-induced phosphorylated p46 JNK (JNK1) levels compared with those in the unphosphorylatable HSP20 overexpressed cells. On the other hand, it is firming established that the effects of TGF-α are exerted through binding EGFR [32]. However, there were no significant differences in the TGF-α-induced phosphorylation of EGFR between the SA-HSP20 transfected HuH7 cells and the SD-HSP20 transfected cells. Taking our findings into account as a whole, it is most likely that phosphorylated HSP20 in HCC cells suppresses the TGF-α-induced cell migration and invasion due to inhibiting the JNK pathway. In addition, it is probable that the effect of phosphorylated HSP20 is exerted at the point between EGFR and JNK in HCC cells. Further investigations are required to elucidate the exact mechanism of phosphorylated HSP20 underlying the suppression of HCC migration and the invasion.

In conclusion, our present findings strongly suggest that phosphorylated HSP20 suppresses TGF-α induced migration of HCC cells and the invasion. HSP20 phosphorylation might provide a new aspect of HCC and could be a novel therapeutic strategy.
